# Development of Adjuvant-Free Bivalent Food Poisoning Vaccine by Augmenting the Antigenicity of *Clostridium perfringens* Enterotoxin

**DOI:** 10.3389/fimmu.2018.02320

**Published:** 2018-10-09

**Authors:** Hidehiko Suzuki, Koji Hosomi, Ayaka Nasu, Masuo Kondoh, Jun Kunisawa

**Affiliations:** ^1^Laboratory of Vaccine Materials and Laboratory of Gut Environmental System, National Institutes of Biomedical Innovation, Health and Nutrition (NIBIOHN), Ibaraki, Japan; ^2^Graduate School of Pharmaceutical Sciences, Osaka University, Suita, Japan; ^3^International Research and Development Center for Mucosal Vaccines, The Institute of Medical Sciences, The University of Tokyo, Tokyo, Japan; ^4^Department of Microbiology and Infectious Diseases, Kobe University Graduate School of Medicine, Kobe, Japan; ^5^Graduate School of Medicine and Graduate School of Dentistry, Osaka University, Suita, Japan

**Keywords:** vaccine, food poisoning, *Clostridium perfringens* enterotoxin, cholera toxin, mucosal immunity

## Abstract

*Clostridium perfringens* enterotoxin (CPE) is a common cause of food poisoning and hyperkalemia-associated death. Previously, we reported that fusion of pneumococcal surface protein A (PspA) to C-terminal fragment of CPE (C-CPE) efficiently bound mucosal epithelium so that PspA-specific immune responses could be provoked. In this study, we found that fusion of C-CPE with PspA augmented the antigenicity of C-CPE itself. These findings allowed us to hypothesize that fusion of C-CPE and another food poisoning vaccine act as a bivalent food poisoning vaccine. Therefore, we constructed an adjuvant-free bivalent vaccine against CPE and cholera toxin (CT), which is a major food poisoning in developing country, by genetically fusing CT B subunit to C-CPE. Because of the low antigenicity of C-CPE, immunization of mice with C-CPE alone did not induce C-CPE-specific immune responses. However, immunization with our vaccine induced both C-CPE- and CT-specific neutralizing antibody. The underlying mechanism of the augmented antigenicity of C-CPE included the activation of T cells by CTB. Moreover, neutralizing antibodies lasted for at least 48 weeks and the quality of the antibody was dependent on the binding activity of CTB–C-CPE to its receptors. These findings suggest that our fusion protein is a potential platform for the development of an adjuvant-free bivalent vaccine against CPE and CT.

## Introduction

Food poisoning is caused by intake of food or water contaminated with pathogens such as bacteria, viruses, parasites, and toxins. The World Health Organization estimates that each year 600 million people worldwide contract food poisoning and 420,000 people die from food poisoning-associated causes (1). Food poisoning is also associated with considerable economic loss [$365 million/year in medical costs for *Salmonella* spp. infections alone in the US (2)]. Despite the seriousness of these problems, effective vaccines against food poisoning are yet to be developed.

*Clostridium perfringens* is a spore-forming bacterium distributed in soil, sewage, and food, and in animals and humans, that is a frequent cause of food poisoning (>4 million cases worldwide/year) and occasionally death (3). *C. perfringens*-associated food poisoning causes an estimated economic burden of $382 million per year in the US (4). The number of food poisoning cases caused by *C. perfringens* has not changed in recent years (5). *C. perfringens* produces four main toxins and one enterotoxin (CPE) (6). Strains of *C. perfringens* are classified into five types based on their toxin production (6). *C. perfringens* type A produces CPE, and the symptoms of food poisoning associated with ingestion of CPE develop 8 to 18 h after intake of contaminated food (6). *C. perfringens* is heat-resistant and produces CPE during spore-forming (7), which means it cannot be killed, or its pathogenicity reduced, by cooking. Therefore, a vaccine against *C. perfringens*-associated food poisoning is needed.

CPE is a single, 319-amino acid polypeptide comprising two domains (8, 9). Its C-terminus (C-CPE) mediates its binding to claudin proteins found in the tight junctions between epithelial cells (9). Within the claudin protein family, CPE binds with high affinity to claudin-3 and -4 and with low affinity to claudin-6, -7, -8, and -14 (10, 11). Once bound, the CPE–claudin complex polymerizes via the N-terminus of CPE to form a pore in the epithelial membrane that increases membrane permeability to Ca^2+^ ion and fluids, leading to the development of diarrhea (12). CPE also induces histological damage to the intestinal epithelium, including villus shortening, epithelial necrosis, and desquamation, which allows CPE to enter the bloodstream and travel to the liver where it binds to claudin-3 and induces potentially life-threatening hyperkalemia (13).

One strategy to prevent toxin-mediated pathogenesis in the intestine is to block toxin–receptor binding by inducing neutralizing secretory IgA, which is the primary effector molecule in the intestinal lumen (14). The induction of antigen-specific IgA antibody responses against oral vaccines is mediated by gut-associated lymphoid tissue (GALT), which contains various immune cells (e.g., B cells, T cells, and dendritic cells) (14). Exogenous antigens are transported from the lumen into GALT by antigen-sampling M cells and dendritic cells, which promote B-cell IgA class-switching and T-cell activation. Upon emigration of IgA-committed B cells from GALT, they traffic to the intestinal lamina propria where they further differentiate into IgA-producing plasma cells. IgA is transported by polymeric immunoglobulin receptors expressed on epithelial cells into the intestinal lumen, where it functions as secretory IgA and inhibits toxin–receptor binding. Some currently licensed oral vaccines against food poisoning (e.g., the cholera vaccines Dukoral and Shanchol) were developed with this underlying strategy (15).

Blocking the binding of CPE to claudins is a possible target for the development of an oral vaccine against CPE-mediated food poisoning. It has been reported that anti-C-CPE monoclonal antibody, which recognizes active site for claudin binding, has neutralizing activity against CPE, but anti-C-CPE monoclonal antibody, which could not recognize active site, fails to neutralize CPE. Therefore, antibody which recognizes active site for claudin binding is important in the protection against CPE (16). However, this domain is lower antigenic domain than the other domains (17). Therefore, it is difficult to induce sufficient immune responses for neutralization against CPE using C-CPE alone.

These features allowed us to employ C-CPE as drug and vaccine delivery system (18, 19). As a vaccine delivery, we previously reported that C-CPE effectively delivered genetically fused antigen to mucosal epithelium, and induced immune responses against fused antigen (19, 20). In this study, we unexpectedly found that C-CPE genetically fused with antigen augmented antigenicity of C-CPE itself and therefore C-CPE-specific immune responses were induced together with immune reposes against fused antigen. In the present study, we used this knowledge to develop an adjuvant-free bivalent vaccine against CPE and cholera toxin (CT).

## Materials and methods

### Mice

Female BALB/c mice (age, 7 weeks) were purchased from CLEA, Inc. (Tokyo, Japan). Mice were housed with a 12-h light/12-h dark cycle and were allowed free access to food and water. All experiments and protocols were approved by the Animal Care and Use Committee of the National Institutes of Biomedical Innovation, Health and Nutrition (approval no. DS27-48R10), and conducted in accordance with the guideline of the Animal Care and Use Committee of National Institutes of Biomedical Innovation, Health and Nutrition.

### Cell culture

Parent and claudin-4-expressing mouse fibroblast cell lines (L cells) were kindly provided by Dr. S. Tsukita (Kyoto University, Kyoto, Japan) (11). Both cell lines were cultured in Dulbecco's Modified Eagle's medium supplemented with 10% fetal bovine serum in a 5% CO_2_ atmosphere at 37°C. Parent and claudin-4-expressing human sarcoma cell lines (HT1080 cells) (ATCC, Virginia, USA) were cultured in Dulbecco's Modified Eagle's medium supplemented with 10% fetal bovine serum in a 5% CO_2_ atmosphere at 37°C (21). African Green Monkey kidney normal cells (Vero cells) (JCRB Cell Bank, Osaka, Japan) were cultured in Dulbecco's Modified Eagle's medium supplemented with 10% fetal bovine serum in a 5% CO_2_ atmosphere at 37°C.

### Preparation of recombinant proteins

pET16b plasmids encoding C-CPE were prepared as previously described (22). pET16b-PspA-C-CPE was prepared as previously described (20) and modified by means of polymerase chain reaction (PCR) (forward primer: 5′-ATAGAAAAAGAAATCCTTGATTTAGCTGCT-3′, reverse primer: 5′-CTCGAATCCTCCAGATCCTCC-3′).

CTB cDNA was amplified by means of PCR (forward primer: 5′-ca*ggtacc*acacctcaaaatattact-3′, *Kpn*I site is underlined; reverse primer: 5′-a*gaattc*ttaatttgccatactaattgc-3′, *Eco*RI site is underlined). pCold II DNA (Takara, Shiga, Japan) and CTB PCR products were digested with *Kpn*I and *Eco*RI. The CTB fragment was inserted into pCold II DNA to yield pCold II-CTB.

CTB–C-CPE cDNA was amplified by means of PCR to yield pCold II- CTB–C-CPE by using a mutagenesis PCR kit (Toyobo, Osaka, Japan) (forward primer: 5′-ATAGAAAAAGAAATCCTTGATTTAGCTGC-3′, reverse primer: 5′-CTCGAATCCTCCAGATCCTCC-3′). CTB–C-CPE mutant cDNA was amplified by means of PCR to yield pCold II-CTB Y12D–C-CPE (forward primer: 5′-GATCACAACACACAAATACATACGC-3′; reverse primer: 5'-TTCTGCACACAAATCAGTAATATTTTG-3'), pCold II-CTB–C-CPE Y306A/L315A (forward primer 1: 5′-gctagtggaaattacccttattcaa-3′, reverse primer 1: 5′-tgatgaattagctttcattacaagaaca-3′; forward primer 2: 5′-gcatttcaaaaattttaagaatt-3′, reverse primer 2: 5′-tattgaataagggtaatttccact-3′), and pCold II-CTB Y12D–C-CPE Y306A/L315A (forward primer 1: 5′-GATCACAACACACAAATACATACGC-3′, reverse primer 1: 5′-TTCTGCACACAAATCAGTAATATTTTG-3′; forward primer 2: 5′-gctagtggaaattacccttattcaa-3′, reverse primer 2: 5′-tgatgaattagctttcattacaagaaca-3′; forward primer 3: 5′-gcatttcaaaaattttaagaatt-3′, reverse primer 3: 5′-tattgaataagggtaatttccact-3′).

To obtain recombinant protein, pET16b plasmids were transformed into *Escherichia coli* strain BL21 (DE3) (Toyobo) and pCold II plasmids were transformed into pG-Tf2/*E. coli* strain BL21 (DE3) (Takara). To induce the production of recombinant protein, isopropyl-D-thiogalactopyranoside (Nacalai Tesque, Kyoto, Japan) was added to the culture medium. The culture pellet was sonicated in buffer A (10 mM Tris–HCl [pH 8.0], 400 mM NaCl_2_, 5 mM MgCl_2_, 0.1 mM phenylmethylsulfonyl fluoride, 1 mM 2-mercaptoethanol, and 10% glycerol). The supernatant was loaded onto a HiTrap HP column (GE Healthcare, Pittsburgh, Pennsylvania, USA). Recombinant protein was eluted with buffer A containing 100–500 mM imidazole. The solvent was exchanged with phosphate-buffered saline (PBS) by using a PD-10 column (GE Healthcare). The concentration of recombinant protein was measured by using a BCA Protein Assay Kit (Life Technologies, Carlsbad, California, USA). The purity of eluted protein was confirmed by using a NuPAGE electrophoresis system (Life Technologies) followed by staining with Coomassie Brilliant Blue. CTB, C-CPE, or CTB–C-CPE was biotinylated by using a biotinylation kit (Thermos Fisher Scientific, Waltham, Massachusetts, USA). For the T-cell proliferation assay, endotoxin was removed from the recombinant protein by using an endotoxin removal kit (Generon, Slough, UK). Endotoxin contaminants in the recombinant protein were measured using ToxinSensor™ Chromatogenic LAL Endotoxin Assay Kit (GenScript, New Jersey, USA). Recombinant protein contained around 300 EU/ml, and recombinant protein treated with endotoxin removal kit contained <5 EU/ml.

### Flow cytometric analysis

Mouse claudin-4-expressing L cells and human claudin-4-expressing HT1080 cells were incubated with recombinant protein for 1 h at 4°C. The cells were washed with 2% newborn calf serum in PBS and incubated with mouse anti-histidine (His) tag antibody (clone J099B12; BioLegend, San Diego, California, USA) for 1 h at 4°C. After washing with 2% newborn calf serum in PBS, the cells were incubated with fluorescein isothiocyanate-labeled goat anti-mouse IgG1 antibody (clone RMG1-1; BioLegend) for 30 min at 4°C. After washing with 2% newborn calf serum in PBS, the cells were stained with 7-aminoactinomycin D (BioLegend) for 10 min at 4°C. The cells were washed with 2% newborn calf serum in PBS, and then analyzed by means of flow cytometry (MACSQuant; Miltenyi Biotec, Auburn, California, USA).

### Histological analysis

Peyer's patches isolated from the mice were embedded in Tissue-Tek optimal cutting temperature compound (Sakura Finetek Japan, Tokyo, Japan) and cut into 6-μm sections by using a cryostat. Sections were fixed in 100% acetone for 1 min at 4°C. To prevent non-specific binding, sections were treated with 2% fetal calf serum in PBS for 30 min at room temperature. Sections were washed with PBS and incubated with biotinylated-CTB, -C-CPE, or -CTB–C-CPE at 4°C overnight. After washing with PBS, sections were stained with Alexa Fluor 546-conjugated streptavidin for 30 min at room temperature. After washing with PBS, sections were stained with 4′,6-diamidino-2-phenylindole (DAPI). The sections were then washed with PBS, mounted in Fluoromount (Diagnostic BioSystems, California, USA), and observed under a fluorescence microscope (BZ-9000; Keyence, Osaka, Japan).

Surgically constructed intestinal loop was fixed in 4% paraformaldehyde, embedded in Tissue-Tek optimal cutting temperature compound (Sakura Finetek Japan), and cut into 6-μm sections by using a cryostat. Sections were stained with hematoxylin for 10 min and eosin for 3 min. The sections were then fixed in ethanol and xylene, mounted in Permount (Thermo Fisher Scientific), and observed under an optical microscope (BZ-9000; Keyence).

### Immunization

Mice were intraperitoneally immunized with C-CPE, a mixture of C-CPE and Imject Alum adjuvant (Thermo Fisher Scientific), or PspA–C-CPE (PspA: 25 μg, C-CPE: 10 μg) once a week for two weeks. One week after the final immunization, serum was collected.

Mice were subcutaneously immunized on the back with vehicle, CTB, C-CPE, a mixture of C-CPE and CT (List Biological Laboratories, Campbell, California, USA), or CTB–C-CPE (CTB: 20 μg, C-CPE: 24 μg, CT: 10 μg). One week after immunization, fasted mice (>12 h~) were orally administered sodium bicarbonate (Otsuka Pharmaceutical, Tokyo, Japan). After 15–20 min, the mice were orally immunized with vehicle, CTB, C-CPE, a mixture of C-CPE and CT, or CTB–C-CPE (CTB: 20 μg, C-CPE: 24 μg, CT: 10 μg) once a week for 3 weeks. One week ~ forty-eight weeks after the final immunization, serum, feces, and intestinal wash were collected. Feces was suspended in PBS (100 mg/mL) and vortexed for 10 min at 4°C. Small intestine was washed with PBS. The samples were centrifuged at 3,000 g for 10 min and the supernatants were collected.

### Measurement of CT-, C-CPE-, or CTB–C-CPE-specific antibody by means of enzyme-linked immune sorbent assay

Ninety-six-well immunoplates were coated with CT (5 μg/mL), C-CPE (10 μg/mL), or CTB–C-CPE (10 μg/mL) and incubated at 4°C overnight. After incubation, the plates were blocked with 1% bovine serum albumin in PBS for 2 h at room temperature. After washing with 0.05% Tween 20 in PBS, two-fold serial diluted serum, fecal extract, or intestinal wash was added to the wells and the plates were incubated for 2 h at room temperature. After washing with 0.05% Tween 20 in PBS, goat anti-mouse IgG, IgG1, IgG2b, or IgG3 conjugated horseradish peroxidase (SouthernBiotech, Birmingham, Alabama, USA) was added to the wells and the plates were incubated for 1 h at room temperature. After washing with 0.05% Tween 20 in PBS, CT-, C-CPE-, or CTB–C-CPE-specific antibodies were detected by using 3,3′,5,5′-tetramethylbenzidine peroxide substrate and measuring absorbance at a wavelength of 450 nm.

### *In situ* loop assay

Mice immunized with vehicle, CTB, C-CPE, a mixture of C-CPE and CT, or CTB–C-CPE were fasted for 24 h. Under anesthesia by isoflurane, the small intestine was withdrawn and a 5- to 9-cm loop was made 6 to 18 cm from the pylorus. CPE (15 μg; Bio Academia, Osaka, Japan) was injected into the loop, and the abdomen was closed. After 90 min, the loop was collected and its weight and length measured.

### *In vitro* CPE neutralizing assay

Vero cells (5.0 × 10^4^ cells) were inoculated into a 96-well plate and cultured in a 5% CO_2_ atmosphere at 37°C. The next day, CPE (0.1 μg) and sera from immunized mice (40 μL) were incubated for 1 h at 37°C. The mixture of CPE and serum was added to the wells and the plates were incubated for 30 min in a 5% CO_2_ atmosphere at 37°C. After gently washing with PBS, Cell Count Reagent SF (Nacalai Tesque) was added to the wells for the detection of living cells. After 1 h, absorbance at a wavelength of 450 nm was measured.

### *In vivo* CPE neutralizing assay

Mice immunized with vehicle, CTB, C-CPE, a mixture of C-CPE and CT, or CTB–C-CPE were intravenously administered CPE (100 μg/kg body weight). After 30 min, symptom of mice was monitored. After 4 h, serum was collected and serum potassium was measured by using a DRI-CHEM NX500 dry chemistry analyzer (Fujifilm, Tokyo, Japan).

### CT–GM1–ganglioside binding neutralizing assay

Ninety-six-well immunoplates were coated with GM1-ganglioside (5 μg/mL) (Sigma-Aldrich, St Louis, Missouri, USA) and incubated at 4°C overnight. After incubation, the plates were blocked with 1% bovine serum albumin in PBS for 2 h at room temperature. After washing with 0.05% Tween 20 in PBS, a mixture of CT (2.5 ng) and serum (4 μl), which had been preincubated for 1 h at 37°C, was added to the wells and the plates were incubated for 2 h at room temperature. After washing with 0.05% Tween 20 in PBS, rabbit anti-CTB antibody (clone ab34992; Abcam, Cambridge, UK) was added to the wells and the plates were incubated for 2 h at room temperature. After washing with 0.05% Tween 20 in PBS, donkey anti-rabbit IgG conjugated horseradish peroxidase (clone Poly4046; BioLegend) was added to the wells and the plates were incubated for 1 h at room temperature. After washing with 0.05% Tween 20 in PBS, the binding of CT to GM1–ganglioside was detected by using 3,3′,5,5′-tetramethylbenzidine peroxide substrate and measuring absorbance at a wavelength of 450 nm.

### *In vivo* CT neutralizing assay

Mice immunized with vehicle, CTB, C-CPE, or CTB–C-CPE were fasted for 12 h and then orally administered sodium bicarbonate. After 15–20 min, the mice were orally challenged with CT (25 μg). After 13–14 h, intestinal fluid volume was measured.

### CD4^+^ T-cell proliferation assay

CD4^+^ T cells were isolated from the spleen of mice immunized with vehicle, C-CPE, or CTB–C-CPE by using anti-CD4 microbeads (Miltenyi Biotec). Antigen-presenting cells were isolated from the spleen of naïve mice and then irradiated (30 Gy) to inhibit growth, but still maintained an ability to process and present antigens to CD4^+^ T cells. The purified CD4^+^ T cells and antigen-presenting cells were cocultured with vehicle, CTB, C-CPE, or CTB–C-CPE (5 μM) in a 5% CO_2_ atmosphere at 37°C. Five days after culture, proliferation was measured by using a CyQUANT Direct Cell Proliferation Assay Kit (Thermo Fisher Scientific).

### Competition binding assay

Biotinylated CTB–C-CPE was pre-incubated with serum from immunized mice for 1 h at 37°C and applied to mouse claudin-4-expressing L cells for 1 h incubation at 4°C. The cells were washed with 2% newborn calf serum in PBS and incubated with Alexa Fluor 488-conjugated streptavidin for 1 h at 4°C. After washing with 2% newborn calf serum in PBS, the cells were stained with 7-aminoactinomycin D (BioLegend) for 10 min at 4°C. The cells were washed with 2% newborn calf serum in PBS, and then analyzed by flow cytometry (MACSQuant).

### Data analysis

Data are presented as mean ± SD or SEM. Statistical analyses were performed by using the non-parametric Mann–Whitney *U*-test (GraphPad Software, San Diego, California).

## Results

### Augmented antigenicity of C-CPE by genetic fusion with vaccine antigen

We previously reported that C-CPE was a useful vehicle for the delivery of a genetically fused antigen [i.e., pneumococcal surface protein A (PspA)] to the mucosal epithelium to induce antigen-specific immune responses without the need for an adjuvant (19, 20). In the present study, we first examined the C-CPE-specific immune responses in mice intraperitoneally immunized with C-CPE, a mixture of C-CPE and alum adjuvant, or PspA–C-CPE (Figure S1). Due to the low antigenicity of C-CPE (17), marginal C-CPE-specific IgG responses were observed in mice intraperitoneally immunized with C-CPE alone or a mixture of C-CPE and alum adjuvant. In contrast, high C-CPE-specific IgG responses were observed in mice intraperitoneally immunized with PspA–C-CPE. We also confirmed that fusion of C-CPE with other antigens (e.g., ovalbumin) increased the antigenicity of C-CPE (unpublished data). Thus, these results confirm that C-CPE itself has low antigenicity, but that its antigenicity can be increased via the genetic fusion of C-CPE with another antigen.

### Construction of a bivalent vaccine by genetically fusing CTB with C-CPE

We hypothesized that genetic fusion of C-CPE with another antigen could be used to produce a bivalent vaccine. Since CPE is associated with food poisoning, we decided to use another toxin associated with food poisoning, CT, which is produced by *Vibrio cholera* (23). Since it is the B subunit of CT (CTB) that binds to the CT receptor galactosyl-N-acetylgalactosaminyl-(sialyl)-galactosylglucosylceramide (GM1)–ganglioside, we hypothesized that CTB would be a good antigen for the development of a vaccine against CT-mediated diarrhea (23, 24). To construct the bivalent vaccine, we prepared a genetically fused protein in which CTB was fused to C-CPE via its N-terminus (CTB–C-CPE) (Figure S2).

**Figure 1:**
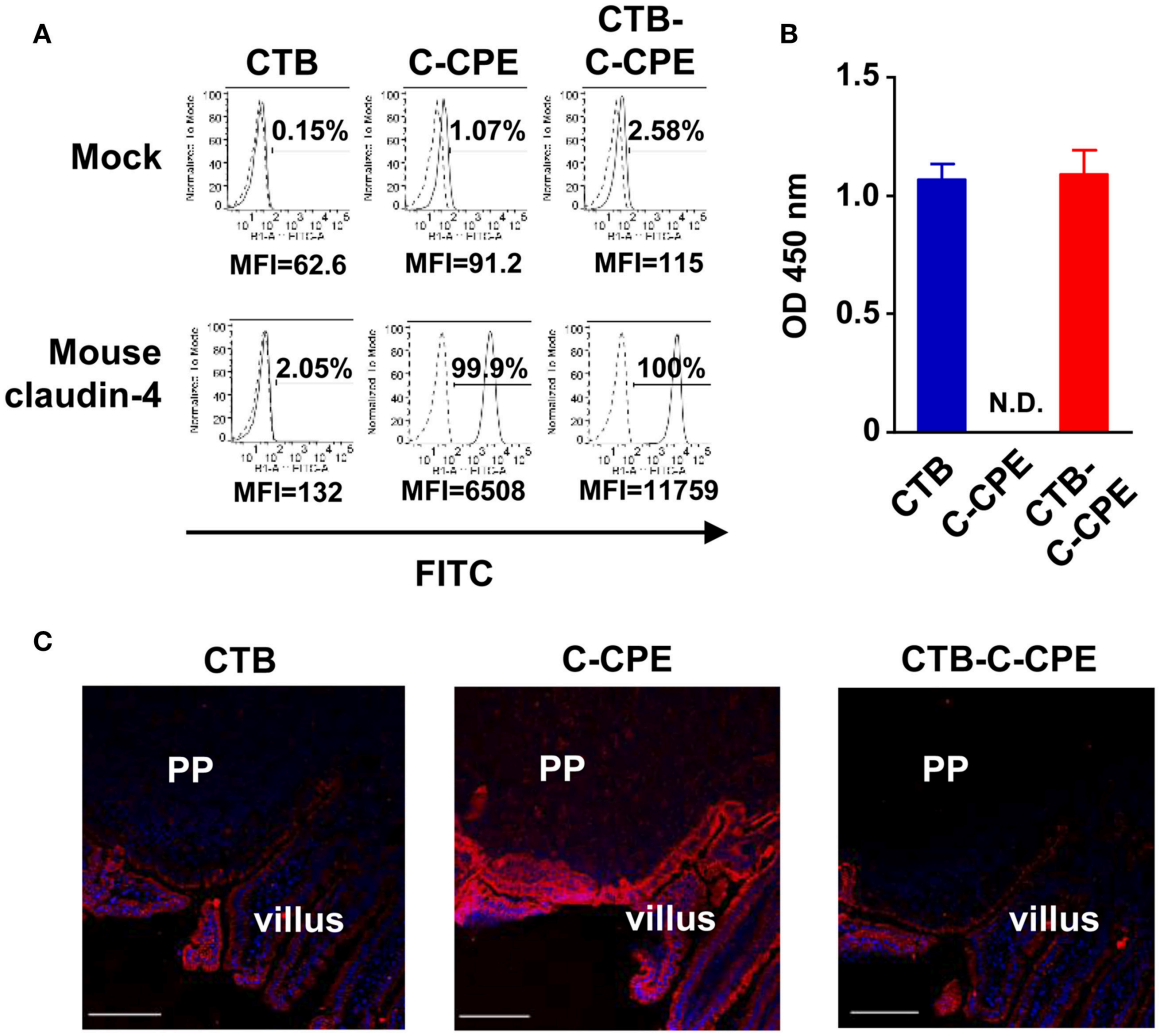
Binding activities of CTB–C-CPE. **(A)** Binding of CTB–C-CPE to claudin-4. Parental and mouse claudin-4-expressing L cells were treated with CTB, C-CPE, or CTB–C-CPE. Receptor binding was detected by using an anti-His tag antibody followed by staining with a fluorescein isothiocyanate (FITC)-labeled secondary antibody. Dashed histograms represent control experiments, and lined histogram is CTB, C-CPE, or CTB–C-CPE. Cells were analyzed by flow cytometry and recorded at least 10,000 cells. Number indicates frequency of FITC-positive cells. MFI, mean fluorescent intensity. Similar results were obtained from two separate experiments. **(B)** Binding of CTB–C-CPE to GM1–ganglioside. Ninety-six-well immunoplates were coated with GM1–ganglioside and then CTB, C-CPE, or CTB–C-CPE was added to the wells. The binding of protein to GM1–ganglioside was detected by using anti-His tag antibody followed by a horseradish peroxidase-labeled secondary antibody. N.D., not detected. Data are presented as mean ± SD (*n* = 3/experiment). Similar results were obtained from two separate experiments. **(C)** Binding of CTB–C-CPE to mouse intestinal epithelium. Intestinal sections (6 μm) were fixed in acetone and stained with biotinylated CTB, C-CPE, or CTB–C-CPE and then stained with Alexa Fluor 546-conjugated streptavidin. Red, biotinylated CTB, C-CPE, or CTB–C-CPE; Blue, DAPI. PP, Peyer's patches. Scale bar, 100 μm. Similar results were obtained from two separate experiments.

Next, we checked the binding activity of CTB–C-CPE to the CPE receptor claudin-4. CTB–C-CPE and C-CPE alone both bound to mouse claudin-4-expressing L cells but not to parent L cells not expressing claudin-4 (Figure 1A). Similarly, CTB–C-CPE and C-CPE alone both bound to human claudin-4-expressing HT1080 cells but not to parent HT1080 cells not expressing claudin-4 (Figure S3). Furthermore, CTB–C-CPE and CTB alone bound to GM1–ganglioside, a receptor for CTB, whereas C-CPE did not (Figure 1B). Consistent with the fact that claudin-4 and GM1–ganglioside are expressed on intestinal epithelium (25, 26), CTB–C-CPE bound to epithelial cells in the mouse intestine, including to cells within Peyer's patches, which are a major component of GALT (Figure 1C). Together, these findings indicate that CTB–C-CPE had binding activities that were comparable with those of CTB or C-CPE alone.

### CTB–C-CPE induces antigen-specific antibodies and protective immunity against CPE

**Figure 2:**
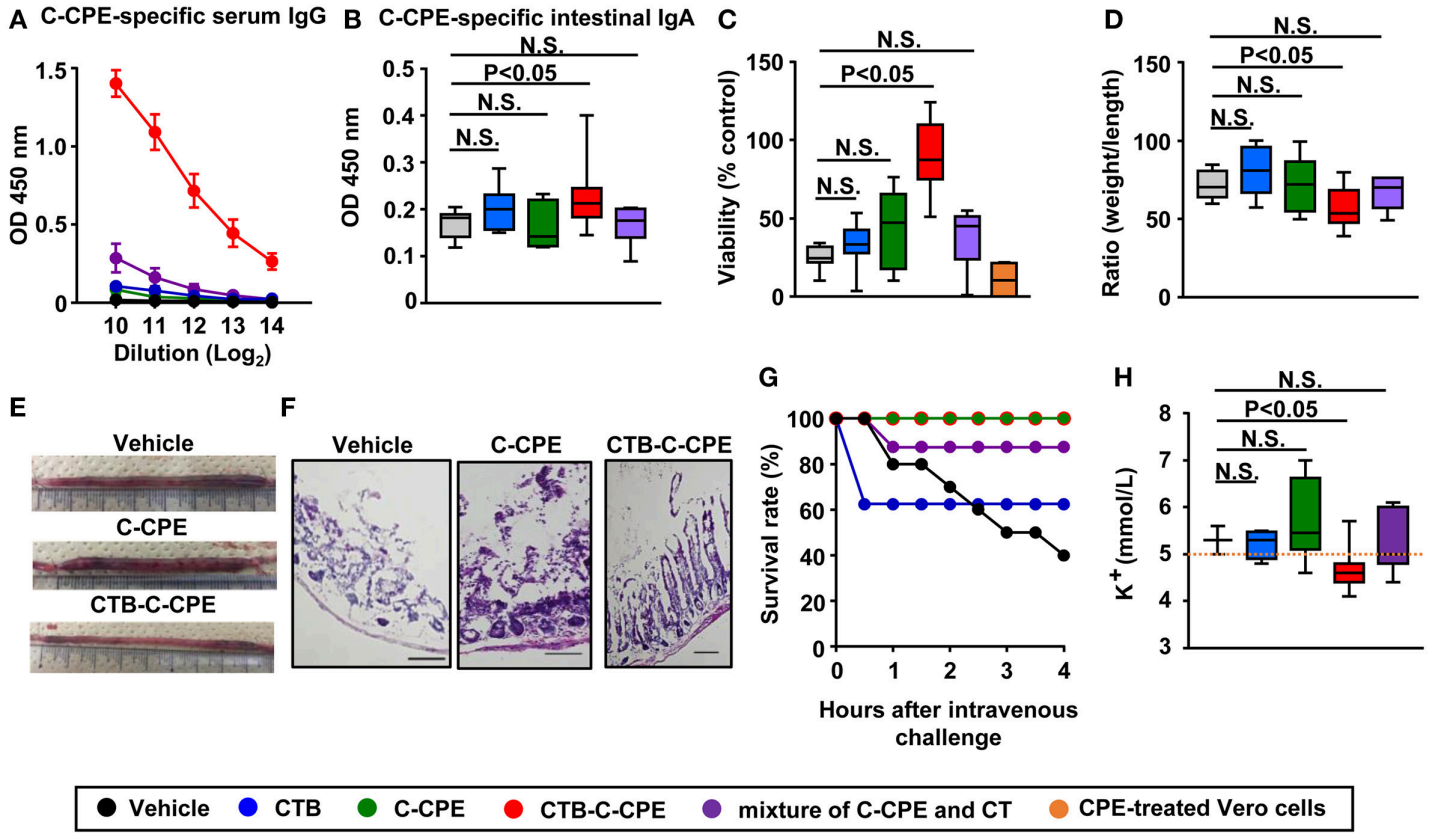
CTB–C-CPE, but not C-CPE or a mixture of C-CPE and CT, induced C-CPE-specific neutralizing immune responses. Production of C-CPE-specific antibodies in the systemic and intestinal compartments. Mice were subcutaneously immunized with vehicle, CTB, C-CPE, CTB–C-CPE, or a mixture of C-CPE and CT (CTB: 20 μg, C-CPE: 24 μg, CT: 10 μg). One week after subcutaneous immunization, mice were orally immunized with vehicle, CTB, C-CPE, CTB–C-CPE, or a mixture of C-CPE and CT once a week for 3 weeks. One week after the final immunization, serum and intestinal wash samples were collected and C-CPE-specific serum IgG **(A)** and intestinal IgA **(B)** levels were determined by means of an enzyme-linked immunosorbent assay. Data are shown as mean ± SEM. N.S., not statistically significant. (*n* = 6–10). **(C)** Neutralizing activity against CPE *in vitro*. Serum from immunized mice was added to Vero cells and cell viability was measured by means of a WST-8 assay. Vero cells treated without CPE were used as controls. (*n* = 4–8). **(D,E)** Protective immunity against CPE-mediated diarrhea. CPE was administered into a surgically constructed intestinal loop (*n* = 6–13). After 90 min, the weight and length of the loop were measured. **(F)** Histological damage by CPE. CPE-treated intestinal loop sections (6 μm; *n* = 5–9) were stained with hematoxylin and eosin. Scale bars, 100 μm. **(G)** Survival in mice administered CPE. Mice were intravenously injected with CPE and survival was monitored. Data were collected from two separate experiments (*n* = 8–12). **(H)** Protection against CPE-mediated hyperkalemia. Mice (*n* = 3–11) were intravenously injected with CPE. After 4 h, serum was collected and the level of potassium was measured. Orange line indicates upper limit of potassium level (5.0 mmol/L). OD, optical density. N.S., not statistically significant. Box plots: Bar represents the median, top is maximum value, bottom is minimum value. Black, vehicle; Blue, CTB; Green, C-CPE; Red, CTB–C-CPE; Purple, mixture of C-CPE, and CT; Orange, positive control (CPE-treated Vero cells). Values were compared by using the non-parametric Mann–Whitney *U*-test.

Induction of neutralizing antibody in the systemic and intestinal compartments is required for the prevention of toxin-mediated pathogenesis (24). In the present study, we used a systemic prime- and oral boost-immunization method because this method induces antibody responses simultaneously in both the systemic and intestinal compartments (27, 28). Mice received initial subcutaneous immunization followed by oral immunization once a week for 3 weeks. One week after the final immunization, serum and intestinal wash samples were collected for the measurement of C-CPE-specific antibody. Immunization with C-CPE alone did not induce any detectable C-CPE-specific antibody responses in either serum or intestinal wash (Figures 2A,B). In contrast, immunization with CTB–C-CPE induced high levels of C-CPE-specific serum IgG and intestinal IgA (Figures 2A,B and Figure S4), and this antibody production was higher than that induced by C-CPE mixed with CT, a strong mucosal adjuvant (29) (Figures 2A,B). These findings indicate that the addition of an adjuvant weakly augmented the antigenicity of C-CPE, but that genetic fusion of C-CPE and CTB strongly increased the antigenicity of C-CPE.

Next, we investigated whether the C-CPE-specific antibodies induced by CTB–C-CPE could provide sufficient protective immunity against CPE-mediated pathogenesis by using Vero cells endogenously expressing claudins and highly sensitive to CPE (30). When the Vero cells were treated with CPE, most of the cells died (Figure 2C, positive control). Similarly, a high degree of cell death was observed in Vero cells treated with CPE after being pretreated with serum from mice immunized with vehicle, CTB, C-CPE, or a mixture of C-CPE and CT. However, when the Vero cells were treated with serum from mice immunized with CTB–C-CPE, CPE-induced cell death was prevented. These results suggest that C-CPE-specific serum antibodies induced by immunization with CTB–C-CPE have neutralizing activity against CPE.

We then examined whether immunization with CTB–C-CPE could prevent the development of CPE-mediated diarrhea in mice. Seven days after the final immunization, mice were administered CPE into a surgically constructed intestinal loop. After 90 min, the weight and length of the loop was measured by using a method previously reported (31). In mice immunized with CTB–C-CPE, the ratio of loop weight to length was significantly smaller compared with that in mice treated with vehicle, which we attributed to inhibition of the development of diarrhea (Figures 2D,E and Figure S5A). Because claudin-4 is highly expressed on villous tips in the bowel, CPE strongly binds to villus tips and causes severe damage, sloughing off or shortening of villi (32–34). Consistent with these previous reports, damage and sloughing off of villi were observed in the intestine of mice immunized with vehicle, CTB, C-CPE, or a mixture of C-CPE and CT and then challenged with CPE. In contrast, no damage was observed in mice immunized with CTB–C-CPE (Figure 2F and Figure S5B). Together, these data indicate that CTB–C-CPE induced protective immunity against CPE-mediated diarrhea and intestinal damage.

Claudin-3 expressed in liver is also a receptor for CPE (33). In severe cases of CPE-mediated food poisoning, CPE enters the bloodstream through damaged parts of the intestine and is transported to the liver where it binds to claudin-3 and increases efflux of potassium from the liver, eventually leading to limb paralysis and occasionally death (13, 34). We therefore checked whether immunization with CTB–C-CPE could protect against CPE-mediated hyperkalemia. Mice immunized with vehicle, CTB, C-CPE, or a mixture of C-CPE and CT exhibited limb paralysis and muscle weakness when intravenously challenged with CPE, and some mice died (Figure 2G and Videos S1–S4). However, mice immunized with CTB–C-CPE did not show any of the symptoms observed in the other mice (Figure 2G and Video S5). As expected, in mice immunized with vehicle, CTB, C-CPE, or a mixture of C-CPE and CT, serum potassium levels were abnormally high (>5.0 mmol/L), whereas in mice immunized with CTB–C-CPE, serum potassium levels remained normal (3.8–5.0 mmol/L), and were significantly lower than mice immunized with vehicle (Figure 2H). Together, these findings indicate that immunization with CTB-C-CPE induced protective immunity against CPE-mediated hyperkalemia.

### Antigenicity of CTB–C-CPE as a vaccine against CT

**Figure 3:**
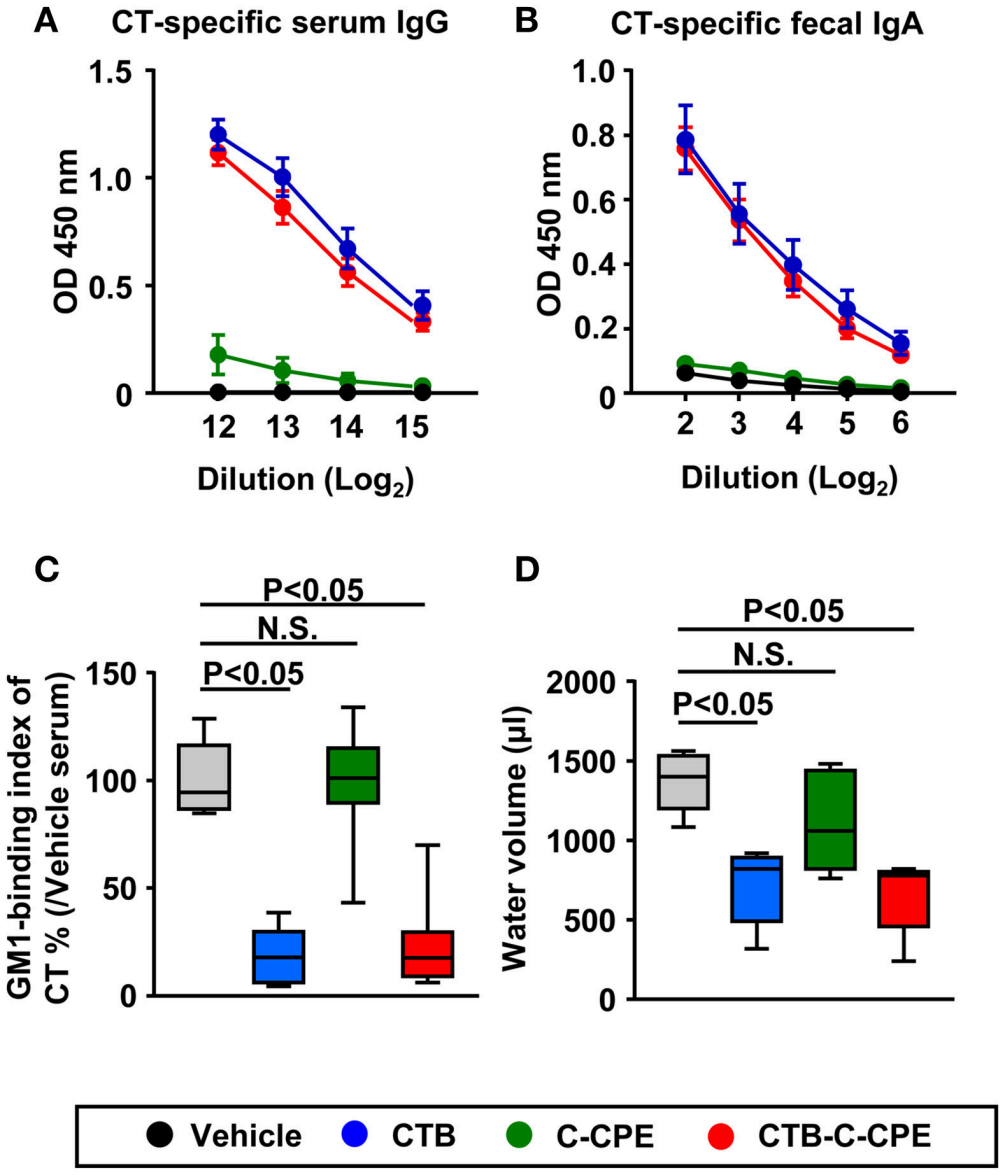
Induction of protective immunity against CT-mediated diarrhea by CTB–C-CPE Mice were subcutaneously immunized with vehicle, CTB, C-CPE, or CTB–C-CPE (CTB: 20 μg, C-CPE: 24 μg). One week after subcutaneous immunization, mice were orally immunized with vehicle, CTB, C-CPE, or CTB–C-CPE once a week for 3 weeks. One week after the final immunization, serum and fecal samples were collected and the levels of CT-specific serum IgG **(A)** and fecal IgA **(B)** were determined by means of an enzyme-linked immunosorbent assay. Data are shown as mean ± SEM. OD, optical density (*n* = 8–9). **(C)** Neutralizing activity against CT–GM1–ganglioside binding. Serum from CTB–C-CPE immunized mice was added to GM1–ganglioside-coated 96-well immunoplates. The binding of CT and GM1–ganglioside was detected by using rabbit anti-CTB antibody followed by a horseradish peroxidase-labeled secondary antibody. Serum from mice immunized with vehicle was used as the control. N.S., not statistically significant. (*n* = 8–9). **(D)** Protective immunity against CT-mediated diarrhea. Eleven days after the final immunization, mice were orally challenged with CT (25 μg). After 13–14 h, intestinal fluid volume was measured. (*n* = 5–6) Box plots: Bar represents the median, top is maximum value, bottom is minimum value. Grey, vehicle; Blue, CTB; Green, C-CPE; Red, CTB–C-CPE. Values were compared by using the non-parametric Mann–Whitney *U*-test. N.S., not statistically significant.

We next investigated whether CTB–C-CPE induced CT-specific antibody responses. Unlike C-CPE, CTB has high antigenicity; therefore, immunization with CTB alone was sufficient to induce CT-specific antibody responses in both the systemic and intestinal compartments (35). Immunization with CTB–C-CPE was sufficient to induce CT-specific antibody responses in both the systemic and intestinal compartments, and these antibody responses were equal to those induced by CTB alone (Figures 3A,B). We also found that, like C-CPE-specific antibody, CT-specific antibody had neutralizing activity; serum from mice immunized with CTB–C-CPE blocked the binding of CT to GM1–ganglioside, and this neutralizing activity was comparable with that of serum from CTB-immunized mice (Figure 3C).

These findings prompted us to investigate whether CTB–C-CPE induced protective immunity against CT-mediated diarrhea. As in the mice administered vehicle, mice immunized with C-CPE alone developed severe diarrhea together with an increased volume of intestinal water (Figure 3D). However, mice immunized with CTB or CTB–C-CPE were protected from developing CT-mediated diarrhea. These findings indicate that CTB–C-CPE induced CT-specific protective immunity against CT-mediated diarrhea.

### Selective T-cell responses against CTB induced by CTB–C-CPE

**Figure 4:**
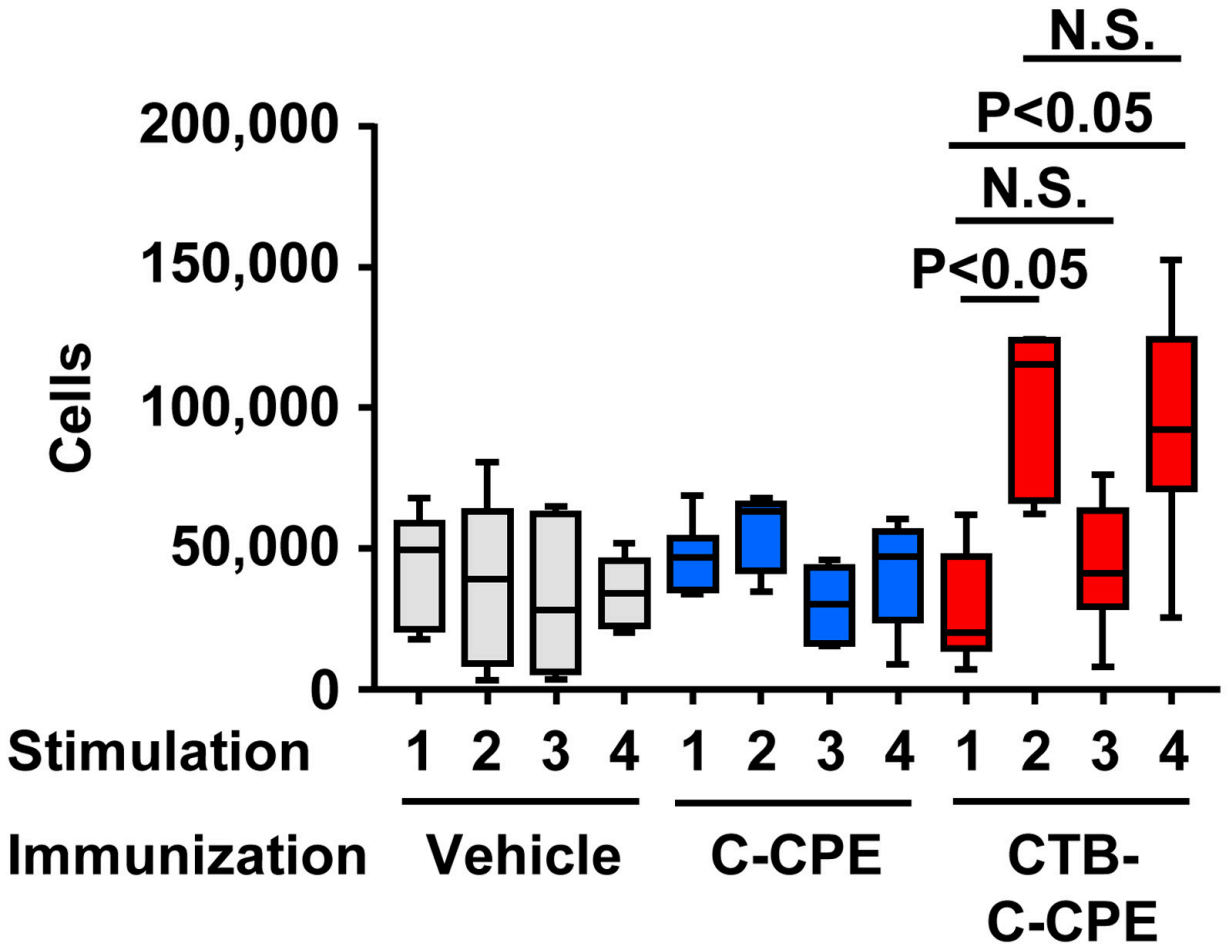
T cells activation by CTB–C-CPE. Mice were subcutaneously immunized with vehicle, C-CPE, or CTB–C-CPE (CTB: 20 μg, C-CPE: 24 μg). One week after subcutaneous immunization, mice were orally immunized with vehicle, C-CPE, or CTB–C-CPE once a week for 3 weeks. One week after the final immunization, splenic CD4^+^ T cells were isolated from the immunized mice. Antigen-presenting cells were isolated from the spleen of naïve mice. Purified CD4^+^ T cells and antigen-presenting cells were stimulated with vehicle, C-CPE, CTB, or CTB–C-CPE. Five days after culture, proliferation was measured. Stimulation 1, vehicle; 2, CTB; 3, C-CPE; 4, CTB–C-CPE (*n* = 6–8). Values were compared by using the non-parametric Mann–Whitney *U*-test.

To investigate the T-cell responses induced by immunization with CTB–C-CPE, we isolated splenic CD4^+^ T cells from mice immunized with vehicle, C-CPE, or CTB–C-CPE and examined their proliferation upon stimulation with antigen in the presence of antigen-presenting cells (Figure 4). No T-cell proliferation was noted in mice immunized with vehicle or C-CPE, irrespective of the antigen used to stimulate the T cells. However, proliferation was induced in T cells isolated from mice immunized with CTB–C-CPE when stimulated with CTB–C-CPE, suggesting that T cells induced by CTB–C-CPE recognized antigens derived from CTB–C-CPE. Indeed, proliferation was also induced in these T cells when they were stimulated with CTB alone but not with C-CPE alone. These results indicate that immunization with CTB–C-CPE induced T cells specifically recognizing antigens derived from CTB.

### CTB–C-CPE induces long-term antibody production

**Figure 5:**
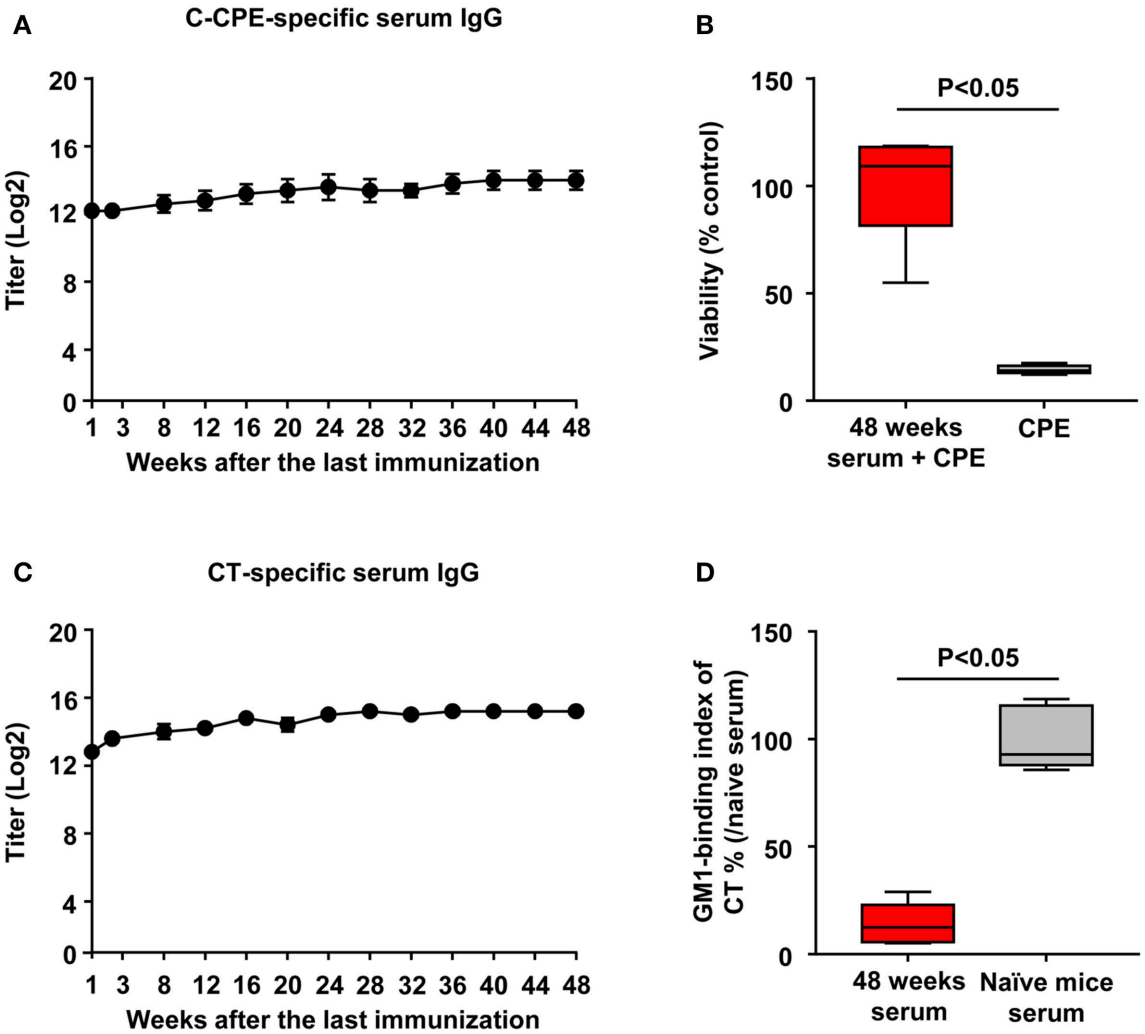
Long-term antibody production by CTB–C-CPE. **(A)** Time course of C-CPE-specific antibody production. One week to forty-eight weeks after the final immunization, serum samples were collected and the levels of C-CPE specific serum IgG was determined by ELISA. Data are shown as mean ± SEM (*n* = 5). **(B)** Neutralizing activity against CPE *in vitro*. CPE and serum from immunized mice were added to Vero cells and cell viability was measured by WST-8 assay. Vero cells treated without CPE were used as controls (*n* = 5). **(C)** Production of CT-specific antibodies. One week to forty-eight weeks after the final immunization, serum samples were collected and the levels of CT-specific serum IgG was determined by ELISA. Data are shown as mean ± SEM (*n* = 5). **(D)** Neutralizing activity against CT–GM1–ganglioside binding. CT and serum from immunized mice were added to GM1–ganglioside-coated 96-well immunoplates. The binding of CT and GM1–ganglioside was detected by using rabbit anti-CTB antibody followed by a horseradish peroxidase-labeled secondary antibody. Serum from naïve mice was used as the control (*n* = 5). Box plots: Bar represents the median, top is maximum value, bottom is minimum value. Values were compared by using the non-parametric Mann–Whitney *U*-test.

To investigate how long the vaccine effects were maintained, we sequentially collect serum for measurement of C-CPE- and CT-specific antibodies. Mice immunized with CTB–C-CPE sustained high level of C-CPE-specific serum IgG for 48 weeks after the last immunization (Figure 5A). Moreover, it exerted neutralizing activity against CPE (Figure 5B). Similarly, high level of CT-specific serum IgG and neutralizing activity against CT were maintained (Figures 5C,D). These findings indicated that CTB–C-CPE could induce long-term neutralizing antibody responses against both CPE and CT.

### Relationship between the binding activity of CTB–C-CPE to its receptors and the quality of the immune responses induced

**Figure 6:**
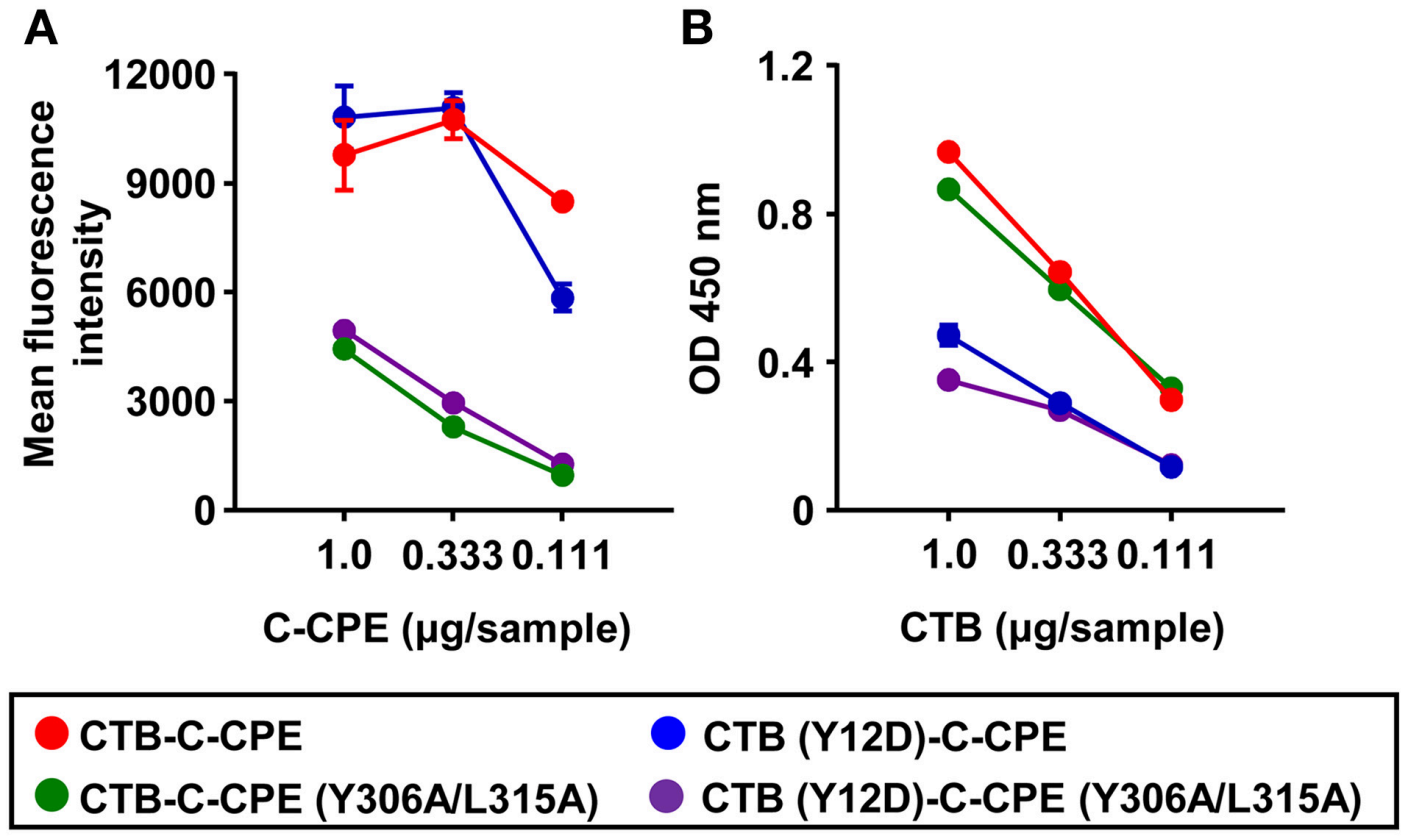
Binding activity of three CTB–C-CPE mutants to claudin-4 and GM1–ganglioside. **(A)** Binding of CTB–C-CPE and three CTB–C-CPE mutants to claudin-4. Parent and mouse claudin-4-expressing L cells were treated with the CTB–C-CPE mutants. Binding was detected by using an anti-His tag antibody followed by staining with fluorescein isothiocyanate (FITC)-labeled secondary antibody (*n* = 3). **(B)** Binding of CTB–C-CPE and three CTB–C-CPE mutants to GM1–ganglioside. Ninety-six-well immunoplates were coated with GM1–ganglioside, and the CTB–C-CPE mutants were then added to the wells. The binding of the CTB–C-CPE mutants to GM1–ganglioside was detected by using anti-His tag antibody followed by horseradish peroxidase-labeled secondary antibody. (*n* = 4). Data are shown as mean ± SD. Red, CTB–C-CPE; Blue, CTB (Y12D)–C-CPE; Green, CTB–C-CPE (Y306A/L315A); Purple, CTB (Y12D)–C-CPE (Y306A/L315A).

Our findings showed that fusion of CTB and C-CPE was crucial for the induction of protective immunity mediated by neutralizing antibody and T cells. Since CTB–C-CPE bound to both claudin-4 and GM1–ganglioside (Figures 1A,B), we examined whether the binding of CTB–C-CPE to claudin-4 or GM1–ganglioside, or both, was essential for the induction of protective immunity. Thirty amino acids in the C-terminus of C-CPE comprise the active site for claudin binding (36). Of these amino acids, we previously demonstrated by using a C-CPE mutant that Y306 and L315 are the most crucial (37, 38). It has also been reported that Y12 of CTB is an important amino acid for the binding of CTB to GM1–ganglioside (39). To test which binding activity was required, we prepared three CTB–C-CPE mutants: CTB (Y12D)–C-CPE, CTB–C-CPE (Y306A/L315A), and CTB (Y12D)–C-CPE (Y306A/L315A) (Figure S6). We confirmed that CTB–C-CPE (Y306A/L315A) and CTB (Y12D)–C-CPE had low binding activity for claudin-4 and GM1–ganglioside, respectively (Figures 6A,B). In addition, we confirmed that CTB (Y12D)–C-CPE (Y306A/L315A) had low binding activity for both claudin-4 and GM1–ganglioside.

**Figure 7:**
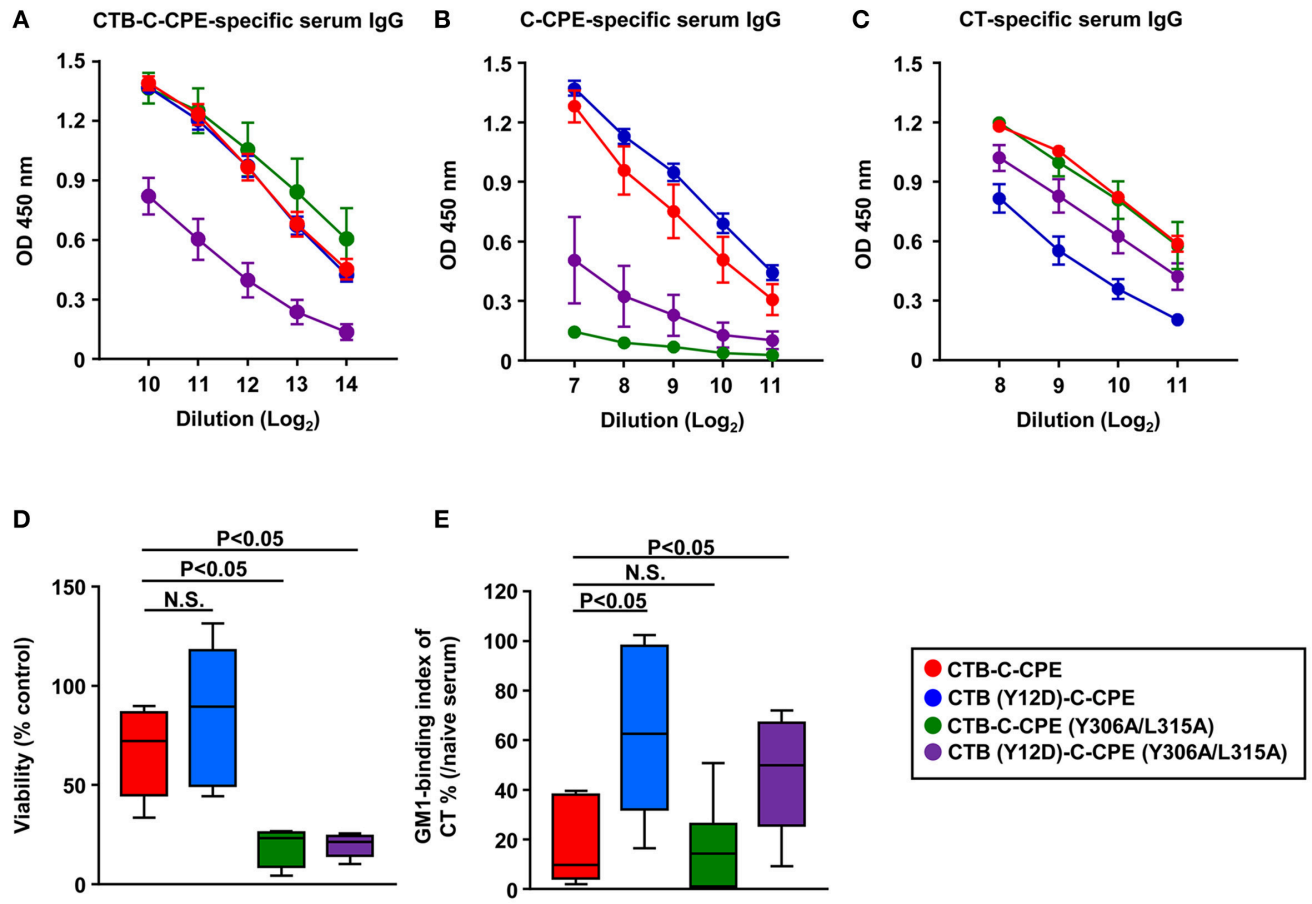
Immune induction and neutralizing activity by three CTB–C-CPE mutants Immune induction by three CTB–C-CPE mutants. Mice were subcutaneously immunized with CTB–C-CPE mutants (CTB: 20 μg, C-CPE: 24 μg). One week after subcutaneous immunization, mice were orally immunized with the CTB–C-CPE mutants once a week for 3 weeks. One week after the final immunization, serum samples were collected and the levels of CTB–C-CPE-specific serum IgG **(A)**, C-CPE-specific serum IgG **(B)**, and CT-specific serum IgG **(C)** were determined by means of enzyme-linked immunosorbent assays. Data are shown as mean ± SEM. OD, optical density (*n* = 4–6). **(D)** Neutralizing activity against CPE. Vero cells treated with serum from immunized mice was added to Vero cells. Cell viability was measured by means of a WST-8 assay. Control was Vero cells not treated with CPE. **(E)** Neutralizing activity against CT–GM1–ganglioside binding. CT treated with serum from immunized mice was added to GM1–ganglioside-coated 96-well immunoplates. Binding of CT and GM1–ganglioside was detected by using rabbit anti-CTB antibody followed by a horseradish peroxidase-labeled secondary antibody. Serum from naïve mice was used in control experiments (*n* = 9–11). Data are shown as mean ± SEM. Box plots: Bar represents median. Red, CTB–C-CPE; Blue, CTB (Y12D)–C-CPE; Green, CTB–C-CPE (Y306A/L315A); Purple, CTB (Y12D)–C-CPE (Y306A/L315A). Values were compared by using the non-parametric Mann–Whitney *U*-test.

We next checked antibody production induced by CTB–C-CPE mutants. The levels of CTB–C-CPE-specific antibodies induced by CTB (Y12D)–C-CPE and CTB–C-CPE (Y306A/L315A) were comparable with parent CTB–C-CPE, but significantly decreased in mice immunized with double mutant CTB (Y12D)–C-CPE (Y306A/L315A) (Figure 7A). We then checked the induction of C-CPE- and CT-specific immune responses by the mutants. The C-CPE-specific immune responses induced by CTB (Y12D)–C-CPE were comparable with those induced by CTB–C-CPE, but those induced by CTB–C-CPE (Y306A/L315A) and the double-mutant CTB (Y12D)–C-CPE (Y306A/L315A) were decreased compared with those induced by CTB–C-CPE (Figure 7B). Similarly, comparable CT-specific immune responses were induced by CTB–C-CPE (Y306A/L315A) and CTB–C-CPE, but those induced by CTB (Y12D)–C-CPE and the double-mutant CTB (Y12D)–C-CPE (Y306A/L315A) were decreased compared with those induced by CTB–C-CPE (Figure 7C). These data indicated that single binding activity of CTB–C-CPE to either claudin-4 or GM1–ganglioside was sufficient for immune induction, but simultaneous deficiency in the binding attenuated immune induction. We found that serum from mice immunized with CTB–C-CPE inhibited the binding of CTB–C-CPE to claudin-4 (Figure S7). The binding of CTB–C-CPE to GM1–ganglioside is likely to be inhibited by anti-CT antibody, implicating that these antibodies influence the efficacy of repeated immunization with CTB–C-CPE.

We then checked the activity of neutralizing antibodies induced by the mutants against CPE and CT. Serum from mice immunized with CTB–C-CPE (Y306A/L315A) or CTB (Y12D)–C-CPE (Y306A/L315A) failed to protect against CPE-induced cell death in Vero cells (Figure 7D). Moreover, serum from mice immunized with CTB (Y12D)–C-CPE or CTB (Y12D)–C-CPE (Y306A/L315A) did not prevent the binding of CT to GM1–ganglioside (Figure 7E). Because antibodies from mice immunized with the mutants showed weak binding activity for their binding sites, it is likely that they did not have neutralizing activity against CPE or CT. These findings indicate that the single binding activity of CTB–C-CPE to either claudin-4 or GM1–ganglioside was sufficient for the induction of immune responses against the whole CTB–C-CPE protein but that the quality of the antibody was dependent on its binding activity to the relevant antigen.

## Discussion

Here, we demonstrated that fusion of an antigen to C-CPE increased the antigenicity of C-CPE. A previous report has indicated that CPE contains at least 10 immunogenic domains, with the N-terminal containing more immunogenic domains than the C-terminal (17). Among these immunogenic domains, the 30 amino acids of the C-terminal were identified as a candidate for vaccine development but this domain has relatively less antigenicity than the other domains. To increase the antigenicity of C-CPE, it has been demonstrated that conjugation of the C-terminal of CPE with thyroglobulin increases its antigenicity, and mice intravenously immunized with this fusion protein showed high serum levels of CPE-neutralizing IgG (40). In addition to thyroglobulin, other proteins such as keyhole limpet hemocyanin and bovine serum albumin have been used as a carrier (41, 42). In the present study, we used CTB as a partner for C-CPE, and it is plausible that CTB acts as carrier for C-CPE. We found that immunization of mice with CTB–C-CPE induced T cells that were stimulated to proliferate by CTB but not C-CPE as we previously reported (19, 20). Therefore, it is possible that C-CPE-specific immune responses could not be induced because C-CPE alone does not activate T cells. However, C-CPE-specific immune responses were induced by utilization of CTB-specific T cells because CTB–C-CPE could induce T cells by CTB. Indeed, a previous study indicated that CTB had one immunodominant epitope and it could activate T cells (43). Moreover, unlike C-CPE, CTB has high antigenicity, and CTB alone could induce CT-specific immune responses because CTB has at least 4 antigenic domains (35, 44). Therefore, CTB is a good partner for C-CPE. In the present study, we used CTB as a carrier for C-CPE and demonstrated that it could be used as an adjuvant-free bivalent vaccine against two toxins that cause foodborne illness by inducing protective immunity against CPE- and CT-mediated pathological responses (i.e., hyperkalemia and diarrhea).

In general, antibodies in the serum and feces are mainly produced by plasma cells at spleen and lamina propria, respectively (45–49). Moreover, T cell responses were also induced, which played an important role in immunoglobulin class switch. It is known that Th1 cytokine supports class switch into IgG2b, and Th2 cytokine supports class switch into IgG1 and IgG3 (50). In this study, we demonstrated that CTB–C-CPE induced C-CPE-specific IgG1, IgG2b, and IgG3. Consistently, we previously reported that fusion of ovalbumin and C-CPE induced Th1- and Th2-type cytokine production (19, 20).

Some enterotoxins including CPE migrate from intestine into systemic compartment, and cause systemic pathological responses. On this point, IgG plays an important role in the protection against systemic pathological responses. IgG binds to toxin in the blood stream, resulted in prevention of the binding between toxin and cellular receptor. IgG also interacts with IgG-Fc receptors (FcγRs), which are expressed on macrophages, and induces phagocytosis and degradation of toxin (51). Indeed, a previous report indicates that the interaction between anthrax toxin-specific IgG1, or IgG2 and FcγR positive cells is important in the protection against toxin because FcγR deficient mice administrated with anthrax toxin-specific IgG1, or IgG2 show no protection (52). Therefore, the induction of C-CPE-specific IgG is important in the protection against CPE-induced systemic pathological response.

IgA plays a pivotal role in intestinal immune responses by binding to and neutralizing toxins. Therefore, the induction of neutralizing IgA is an important part of host protection against CT- and CPE-mediated diarrhea because they are toxin-mediated conditions. Indeed, mice deficient in polymeric immunoglobulin receptor (a key factor in the secretion of IgA into the lumen) immunized with CTB vaccine produce CTB-specific serum IgG, but not fecal IgA, resulting in the development of diarrhea (24). Moreover, mice injected with a mixture of CPE-neutralizing monoclonal antibody and CPE into a surgically created intestinal loop show decreased CPE-mediated histological damage compared with mice injected with CPE (53). Therefore, induction of CT- and CPE-specific IgA is important in suppression of CT- and CPE-mediated diarrhea.

The results of the present study show that the binding of CTB–C-CPE to either claudin-4 or GM1–ganglioside was important for the induction of antibodies to C-CPE or CT. There are several pathways through which these antigen-specific antibody responses could be induced.

In terms of the C-CPE-mediated immune response induction pathway, the main receptor for CTB–C-CPE is claudin-4. We previously showed that claudin-4 was expressed on epithelial cells, including on M cells, of nasopharyngeal-associated lymphoid tissue and Peyer's patches (19, 20, 25, 54). In addition, previous reports have shown that C-CPE-derived-peptide-conjugated particles bind to claudin-4-expressing M cells and induce antigen-specific immune responses (55, 56). Consistent with these previous results, the results of the present study indicated that CTB–C-CPE also bound to cells in the epithelium of Peyer's patches. Since M cells have a pocket structure in the basal membrane where dendritic cells are present, dendritic cells may take up CTB–C-CPE via M cells for presentation to T and B cells for the induction of C-CPE- and CT-specific immune responses (57).

Furthermore, claudin-4 has clathrin-sorting signal sequences in its C-terminal intracellular domain (58, 59). We previously reported that protein synthesis inhibitory factor-fused C-CPE bound to claudin-4 and was internalized (60). Therefore, CTB–C-CPE may bind to claudin-4 expressed on intestinal epithelium cells and then be internalized via clathrin-dependent endocytosis for immune induction.

In addition, there is also a unique antigen sampling mechanism in the intestine in which CD103^+^ dendritic cells located on the interior of Peyer's patches take up antigen by elongating their membrane protrusions into the lumen for antigen sampling (61). CD103^+^ dendritic cells also express claudin-4 as part of tight junctions (62). Therefore, CTB–C-CPE may be taken up directly by the elongated membrane protrusions of CD103^+^ dendritic cells or it may bind to claudin-4 expressed on dendritic cells before being internalized by the cells.

In terms of the pathway for the induction of the CTB-mediated immune response, the main receptor for CTB–C-CPE is GM1–ganglioside. A previous study has indicated that CTB is internalized by clathrin-dependent endocytosis (63). Therefore, one possibility is that CTB–C-CPE binds to GM1–ganglioside expressed on intestinal epithelium cells, and is then internalized via clathrin-dependent endocytosis for immune induction.

Moreover, GM1–ganglioside is also expressed on the epithelium of Peyer's patches, including on M cells (26), and it has been shown that CTB-conjugated vaccines are taken up both by Peyer's patches, through the binding of GM1–ganglioside and CTB, and by dendritic cells (64–66). Therefore, it is possible that CTB–C-CPE is also taken up through these pathways.

Thus, CTB–C-CPE can be taken up into intestinal cells via C-CPE–claudin-4- or CTB–GM1–ganglioside-mediated pathways. Indeed, in the present study we showed that the mutants CTB (Y12D)–C-CPE and CTB–C-CPE (Y306A/L315A) induced immune responses via CTB- or C-CPE-mediated immune induction pathways, depending on whether they were able to bind claudin-4 or GM1–ganglioside. As expected, the double-mutant CTB (Y12D)–C-CPE (Y306A/L315A) showed less immune induction than the other mutants. However, this double mutant did not completely attenuate immune induction. These findings suggest that CTB–C-CPE has dual roles as a vaccine. Firstly, CTB–C-CPE acts as an antigen and second one includes delivery function. We suppose that the former role may be enhanced in the presence of specific antibodies (e.g., Fc receptor-mediated uptake by antigen presenting cells). On the other hand, the latter role is likely to be attenuated by the specific antibodies. These effects may influence the efficacy of repeated immunization with CTB–C-CPE and therefore we have to design immunization protocol carefully (e.g., administration route, timing, and receptor expression at each tissue).

In the present study, we found that the binding activities of C-CPE–claudin-4 and CTB–GM1–ganglioside were crucial for the induction of antigen-specific antibodies with neutralizing activity. Indeed, a previous report indicated that mice immunized with a tetanus toxin heavy-chain mutant that lacked binding activity to its receptor exhibited low levels of anti-tetanus toxin antibody, including neutralizing antibody, because the mutant lacked an essential protective epitope, and binding activity (67). Thus, the present results provide additional evidence that for protective vaccine antigens, the receptor binding domain must be maintained to ensure the induction of functional antibodies.

The order in which the proteins are fused is also important for maximizing the induction of immune responses by fusion proteins. A previous study indicated that a fusion protein comprising CTB and the rotavirus globular protein VP8-1 (CTB–VP8-1) showed greater binding affinity for GM1–ganglioside compared with the reverse fusion protein, VP8-1–CTB. Moreover, CTB–VP8-1 elicited a higher titer of neutralizing antibodies against rotavirus and showed a higher antigenicity than did VP8-1–CTB. Because the N-terminus of CTB contains the GM1–ganglioside-binding residues, and N-terminus of VP8-1 extends from globular domain and N-terminus is flexible domain of VP8-1. Thereby, it is plausible that the binding activity, antigenicity and conformation of the antigens were disrupted more in VP8-1–CTB compared with in CTB–VP8-1 (68). Similarly, for CTB–C-CPE, the N-terminus of CTB and C-terminus of C-CPE are important for their binding to their respective receptors (36–39). Therefore, we considered the linking of CTB to the N-terminus of C-CPE to be a reasonable order for maximizing receptor binding and the induction of neutralizing immune responses.

For clinical application of CTB–C-CPE, the expression of claudin-4 and GM1–ganglioside in the human intestine is important. Claudin-4 is expressed on human intestinal epithelial cells including M cells (69, 70). We previously found that C-CPE had high affinity for human claudin-4 (22), and in the present study, we found that CTB–C-CPE bound to human claudin-4-expressing cells. In addition, GM1–ganglioside is expressed on human intestinal epithelial cells, including M cells, and CTB binds to human GM1–ganglioside (71, 72). These results, therefore, imply that CTB–C-CPE will bind to claudin-4 and GM1–ganglioside expressed on human intestinal epithelium, including on M cells.

The safety of CTB–C-CPE is also important for its clinical application. Previously, we reported that mice intravenously immunized with C-CPE showed no hepatic or renal side effects (73), even though claudin-3 and−4 are expressed in liver and kidney (74). This is likely because C-CPE lacks the N-terminus of CPE and therefore it does not polymerize to form pores (12). Like CPE, CT has two domains, with the A subunit, not the B subunit, increasing the ion and water permeability of epithelial membranes, which leads to the development of diarrhea (23). Therefore, CTB is safe in this respect (75). Although safety of CTB–C-CPE should be examined carefully in the future study, it is likely that CTB–C-CPE has no cytotoxicity.

Long-term immune induction is also important factor for the vaccine development and we noted that CTB–C-CPE could induce long-term protective immunity against both CPE and CT. A previous study indicated that antigen-specific antibody could be detected longer than 1 year together with long-lived plasma cells even if half-life of IgG is generally considered to be 21 days (76, 77). Therefore, long-lived plasma cells were induced by CTB–C-CPE.

In summary, genetic fusion of CTB to C-CPE increased the antigenicity of C-CPE. The fusion protein induced both C-CPE- and CT-specific immune responses in the intestinal and systemic immune compartments in mice, suggesting that CTB–C-CPE could be potentially useful for the development of a bivalent vaccine against CPE- and CT-mediated food poisoning.

## Ethics statement

All experiments and protocols were approved by the Animal Care and Use Committee of the National Institutes of Biomedical Innovation, Health and Nutrition (approval no. DS27-48R10), and conducted in accordance with the guideline of the Animal Care and Use Committee of National Institutes of Biomedical Innovation, Health and Nutrition. This guideline has been established based on Act on Welfare and Management of Animals, and Standards Relating to the Care and Keeping and Reducing Pain of Laboratory Animals, established by the Ministry of the Environment in Japan, and Basic policies for the conduct of animal experimentation in the Ministry of Health, Labor and Welfare, established by Ministry of Health, Labor and Welfare in Japan, and Guidelines for Proper Conduct of Animal Experiments, established by Science Council of Japan.

## Author contributions

HS planned and performed the experiments, analyzed the data, and wrote the manuscript. KH and AN performed the experiments and analyzed the data. MK provided helpful discussion. JK planned the experiments and wrote the manuscript. All authors approved the final version of the manuscript.

### Conflict of interest statement

The authors declare that the research was conducted in the absence of any commercial or financial relationships that could be construed as a potential conflict of interest.
