# Supplementary material for: Development of Adjuvant-Free Bivalent Food Poisoning Vaccine by Augmenting the Antigenicity of Clostridium perfringens Enterotoxin
Source: Front Immunol. 2018 Oct 9;9:2320. doi: 10.3389/fimmu.2018.02320 (PMC6189403; doi:10.3389/fimmu.2018.02320)
Supplement: Supplementary file 6 [file Presentation_1.pdf]

Supplementary Material

Development of adjuvant-free bivalent food poisoning vaccine by augmenting the antigenicity of *Clostridium perfringens* enterotoxin

Hidehiko Suzuki[1], Koji Hosomi[1], Ayaka Nasu[1], Masuo Kondoh[2], and Jun Kunisawa[1-5*]

1 Laboratory of Vaccine Materials and Laboratory of Gut Environmental System, National Institutes of Biomedical Innovation, Health and Nutrition (NIBIOHN), Ibaraki, Japan

2 Graduate School of Pharmaceutical Sciences, Osaka University, Suita, Japan

3 International Research and Development Center for Mucosal Vaccines, The Institute of Medical Sciences, The University of Tokyo, Tokyo, Japan

4 Department of Microbiology and Infectious Diseases, Kobe University Graduate School of Medicine, Kobe, Japan

5 Graduate School of Medicine and Graduate School of Dentistry, Osaka University, Suita, Japan

[*]Correspondence:
Jun Kunisawa
kunisawa@nibiohn.go.jp

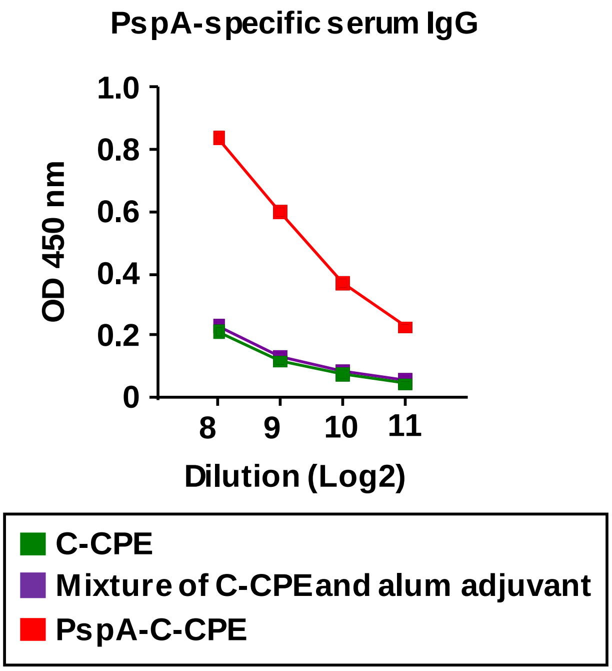

Figure S1 | Fusion of C-CPE and antigen increases the antigenicity of C-CPE. Mice were intraperitoneally immunized with C-CPE, a mixture of C-CPE and alum adjuvant, or PspA–C-CPE (PspA: 25 µg, C-CPE: 10 µg) once a week for two weeks. One week after the final immunization, serum samples were collected and the serum level of C-CPE-specific IgG was determined by means of an enzyme-linked immunosorbent assay. Data are shown as mean ± SEM. OD, optical density. Green, C-CPE; purple, mixture of C-CPE and alum adjuvant; red, PspA–C-CPE. (n=5)

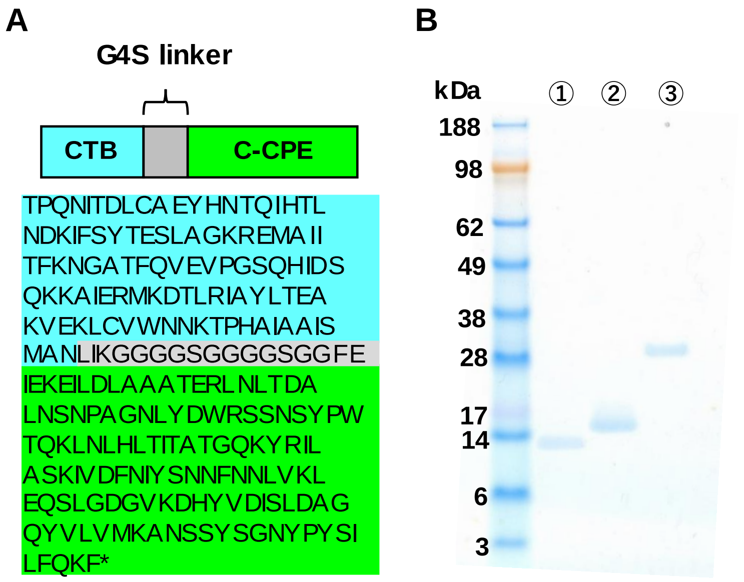

Figure S2 | Construction and preparation of CTB–C-CPE. (A) Schematic illustration and amino acid sequence of CTB–C-CPE. CTB was fused with C-CPE at its N-terminus. A G4S linker was inserted between CTB and C-CPE. **(B)** Purification of recombinant proteins. CTB, C-CPE, and CTB–C-CPE were prepared as His-tagged proteins and purified by means of Ni-affinity chromatography. The purity of the eluted CTB, C-CPE, and CTB–C-CPE was confirmed by using a NuPAGE electrophoresis system followed by staining with Coomassie Brilliant Blue. Lane 1, CTB; lane 2, C-CPE; lane 3, CTB–C-CPE.

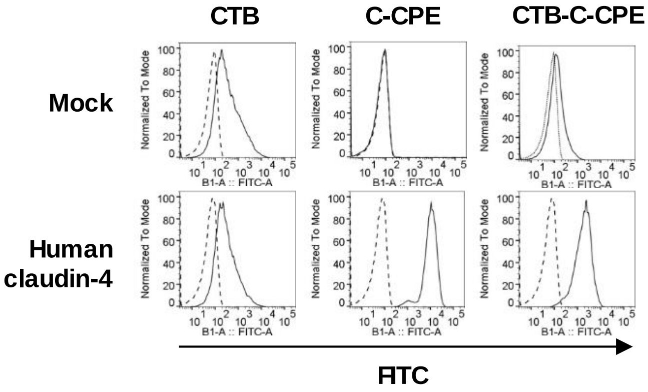

Figure S3 | Binding activity of CTB–C-CPE to human claudin-4. Parent and human claudin-4-expressing HT1080 cells were treated with CTB, C-CPE, or CTB–C-CPE. Receptor binding was detected by using an anti-His tag antibody followed by staining with a fluorescein isothiocyanate (FITC)-labeled secondary antibody. Dashed histograms represent control experiments, and lined histogram is CTB, C-CPE, or CTB–C-CPE.

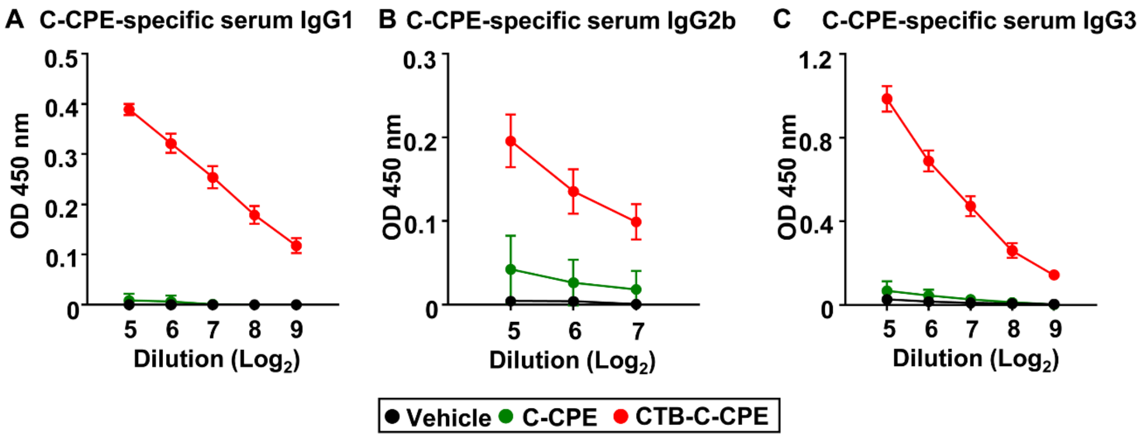

Figure S4 | Analysis of serum C-CPE-specific IgG subclass. Mice were subcutaneously immunized with vehicle, C-CPE, or CTB–C-CPE (CTB: 20 µg, C-CPE: 24 µg). One week after subcutaneous immunization, mice were orally immunized with vehicle, C-CPE, or CTB–C-CPE once a week for three weeks. One week after the final immunization, serum samples were collected and C-CPE-specific serum levels of IgG1 **(A)**, IgG2b **(B)**, and IgG3 **(C)** were determined by means of an enzyme linked immunosorbent assay. Data are shown as mean ± SEM. OD, optical density. Black, C-CPE; Green, mixture of C-CPE and Alum; Red, CTB–C-CPE. (n=3~5)

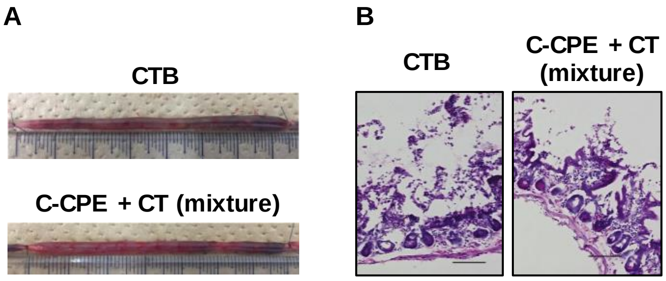

Figure S5 | A mixture of C-CPE and CT did not suppress CPE-induced histological damage to the intestinal epithelium. Mice were subcutaneously immunized with CTB or a mixture of C-CPE and CT (CTB: 20 µg, C-CPE: 24 µg, CT: 10 µg). One week after subcutaneous immunization, mice were orally immunized with CTB or a mixture of CT and C-CPE once a week for three weeks. One week after the final immunization, mice were administered CPE into a surgically constructed intestinal loop **(A)**. CPE-treated intestinal loop sections (6 µm) stained with hematoxylin and eosin **(B)**. Scale bar, 100 µm. (n=5~8)

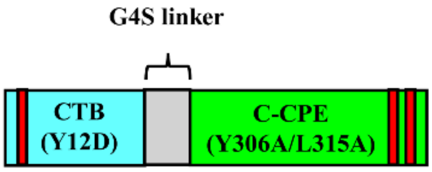

TPQNITDLCAEDHNTQIHTL
NDKIFSYTESLAGKREMAII
TFKNGATFQVEVPGSQHIDS
QKKAIERMKDTLRIAYLTEA
KVEKLCVWNNKTPHAIAAIS
MANLIKGGGGSGGGGSGGFE
IEKEILDLAAATERLNLTDA
LNSNPAGNLYDWRSSNSYPW
TQKLNLHLTITATGQKYRIL
ASKIVDFNIYSNNFNNLVKL
EQSLGDGVKDHYVDISLDAG
QYVLVMKANSSASGNYPYSI
AFQKF*

Figure S6 | Construction of CTB–C-CPE mutants. Schematic illustration and amino acid sequences of three CTB–C-CPE mutants. CTB (Y12D) was fused with C-CPE (Y306A/L315A) at its N-terminus. A G4S linker was inserted between CTB (Y12D) and C-CPE (Y306A/L315A). Red letters indicate substituted amino acids.

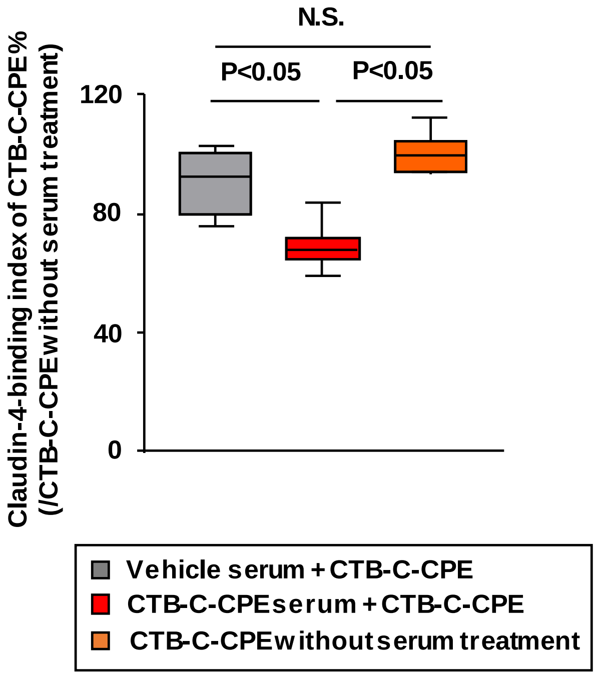

Figure S7 | The binding of CTB–C-CPE to claudin-4 is inhibited by serum from mice immunized with CTB–C-CPE. Mouse claudin-4-expressing L cells were treated with a mixture of biotinylated CTB–C-CPE and serum, which had been preincubated for 1 h at 37°C, for 1 h at 4°C. Receptor binding was detected by using Alexa Fluor 488-conjugated streptavidin. Cells were analyzed by flow cytometry and recorded at least 10000 cells (n = 6–10). Box plots: Bar represents the median, top is maximum value, bottom is minimum value. CTB–C-CPE without serum treatment was used as the control. Values were compared by using the non-parametric Mann–Whitney *U* test. N.S., not statistically significant.

Video S1 | Symptoms of hyperkalemia in mice immunized with vehicle followed by CPE challenge. Mice were subcutaneously immunized with vehicle. One week after subcutaneous immunization, the mice were orally immunized with vehicle once a week for three weeks. One week after the final immunization, the mice were intravenously injected with CPE. After 30 min, the presence of symptoms of hyperkalemia was assessed. Video S1 shows one of 10 mice immunized with vehicle; all mice in this group exhibited comparable symptoms.

Video S2 | Symptoms of hyperkalemia in mice immunized with CTB followed by CPE challenge. Mice were subcutaneously immunized with CTB (CTB: 20 μg). One week after subcutaneous immunization, the mice were orally immunized with CTB once a week for three weeks. One week after the final immunization, the mice were intravenously injected with CPE. After 30 min, the presence of symptoms of hyperkalemia was assessed. Video S2 shows one of eight mice immunized with CTB; all mice in this group exhibited comparable symptoms.

Video S3 | Symptoms of hyperkalemia in mice immunized with C-CPE followed by CPE challenge. Mice were subcutaneously immunized with C-CPE (C-CPE: 24 μg). One week after subcutaneous immunization, the mice were orally immunized with C-CPE once a week for three weeks. One week after the final immunization, the mice were intravenously injected with CPE. After 30 min, the presence of symptoms of hyperkalemia was assessed. Video S3 shows one of eight mice immunized with C-CPE; all mice in this group exhibited comparable symptoms.

Video S4 | Symptoms of hyperkalemia in mice immunized with a mixture of C-CPE and CTB followed by CPE challenge. Mice were subcutaneously immunized with a mixture of C-CPE and CT (C-CPE: 24 μg, CT: 10 μg). One week after subcutaneous immunization, the mice were orally immunized with a mixture of C-CPE and CT once a week for three weeks. One week after the final immunization, the mice were intravenously injected with CPE. After 30 min, the presence of symptoms of hyperkalemia was assessed. Video S4 shows one of eight mice immunized with a mixture of C-CPE and CTB; all mice in this group exhibited comparable symptoms.

Video S5 | Symptoms of hyperkalemia in mice immunized with CTB–C-CPE followed by CPE challenge. Mice were subcutaneously immunized with CTB–C-CPE (CTB: 20 μg, C-CPE: 24 μg). One week after subcutaneous immunization, the mice were

orally immunized with CTB–C-CPE once a week for three weeks. One week after the final immunization, the mice were intravenously injected with CPE. After 30 min, the presence of symptoms of hyperkalemia was assessed. Video S5 shows one of 12 mice immunized with CTB–C-CPE; all mice in this group exhibited comparable symptoms.
